# Art therapy in Alzheimer’s disease. An opportunity of collaboration between intersectoral public and private organizations in the co-design of health and social care services

**DOI:** 10.3389/fpsyt.2023.1198613

**Published:** 2023-11-30

**Authors:** Martina Giusti, Niccolò Persiani

**Affiliations:** Department of Clinical and Experimental Medicine, School of Human Sciences, University of Florence, Florence, Italy

**Keywords:** art therapy, social-health care services, public-private collaboration, co-design, co-responsability

## Abstract

**Introduction:**

The World Health Organization (WHO) has recognized art therapy as an effective supportive mechanism for the maintenance and restoration of mental health. In recent years, art therapy has been integrated in the assistance pathways of older people affected by neurocognitive disorders according to the demonstrated benefits, as no conflicts with pharmacologic treatments and the reduction of anxiety and stress. The shortage of organizational, economic, and professional resources in social-health public organizations does not allow for guaranteeing the provision of these services without the help of the private ones, not exclusively belonging to social-health sector. This research aims to investigate how the collaboration between public and private organizations of different sectors in the co-provision of non-pharmacological approaches guarantees the economic sustainability and the quality improvement of the provided services.

**Methods:**

The Alzheimer Café of Prato was selected as a significant case study.

**Results and Discussion:**

Art therapy programs intended for taking care of older people in the first stages of the Alzheimer’s disease have been developed, planned, supplied, and managed over the years as a result of the integration of resources, ideas, and professionals provided both by public and private sectors, the social-health sector, and cultural organizations.

**Conclusions:**

The peer-to-peer co-responsibility of all organizations (public and private, from the cultural sector as well as the social and health sector) involved in the co-design of art therapy programs, not limiting their actions to only co-financing and/or co-delivery of the service, enabled the achievement of the economic sustainability of the services and the improvement of their quality.

## 1 Theoretical framework

In America, the “art therapy” term was made up by psychoanalytic scholars around the middle of the 20th century, and they prepared different roles of art in psychoanalysis: from art productions as symbolic communication of the unconscious material in a direct, uncensored, and concrete form to art as therapy (1). Art therapy is based on the idea that the creative process for making art or for living with art helps people in the expression, exploration, and understanding of their emotional and psychological spheres (2–4). The quality of the final artistic product is less important than the use of art to help people in expressing themselves (5). Both in America and in Europe, the 1970s through the early 1990s showed the exponential increase of interest on art therapy due to the identification of a broad range of art therapy’s applications with many patient targets (6–8) of all ages (9–11). The same WHO has recognized the fundamental contribution of art in the promotion of wellbeing and correct lifestyles, thereby improving their quality of life. Art therapy is an unconventional tool to overwhelm and resolve critical issues that occur due to both social and health conditions (12, 13), working as a preventive intervention (14).

The most important social-health areas for art therapy application are as follows:

- Psychosocial care is focused on the personal expression of the patients about their physical and physiological difficulties (15–18). Art therapy supports the self-efficacy of patients who can find a new version of themselves after they lose control of their lives due to social and/or health frailty.
- Rehabilitation with the use of art therapy helps the patients to maintain their cognitive and physical abilities or rather slow down their cognitive and physical ability decay (19, 20).
- Health and social benefits from art therapy is complementary to health and social care treatments, increasing the achievement of positive health and social outcomes (21–23).
- Other aspects include the re-authoring of the dominant narrative of the illness or physical disability by the patient, thereby personally describing the own condition (24).

In recent years, the introduction of art therapy in many health and social care settings (i.e., inpatients wards, outpatient services, nursing homes, hospice, rehabilitative centres) has been favoured for its application in humanizing care (25).

Among all possible targets, art therapy is especially used with older people owing to its contribution in healthy aging and its action as a protective factor against the physiological loss of physical and cognitive functions (26). Art therapy as a non-pharmacological approach (27) is especially strongly recommended to older people with complex clinical conditions for the management of mild, moderate, and, sometimes, severe neurological symptoms as are no effective pharmacological treatments to alleviate the disease effects (28, 29). In fact, it avoids interference and lacks side effects due to the consumption of drugs (30, 31). Art therapy is the most widespread treatment for Alzheimer’s disease as it reduces the phenomena of psychosis, wandering, and restlessness, thereby relaxing these patients (32).

Art therapy is a commonly known non-pharmacological treatment option, and art therapy programs require the investment of many resources (fundings, locations, time, and professionals) for the development of personalized interventions (33) to maximize the possible health and social outcomes (34, 35). Finding a solution on how to continue guaranteeing the provision of these expensive health and social care services over time has been a priority in the recent years. In public sector, the resources have been always more limited in the face of the progressive increase in demand due to the ageing of the population and the development of preventive interventions as the ever-increasing costs for the provision of these services due to for their greater complexity (36–38). At the worldwide level, the co-financing of these services by private organizations has been identified as the main solution for guaranteeing not only the provision of integrated health and social care services (39, 40) on account of resource shortage in the public sector but also their economic sustainability and their quality improvement (41, 42). The private sector is not here to replace the services provides by the traditional public sector but rather to collaborate for finding joint solutions through co-funding, and co-provision (43, 44). These studies, however, are focused only on the collaboration between public and private organizations working in the health care and/or in the social care sectors. However, referring to non-pharmacological approaches, the frequency of collaboration between public and private organizations in the education or cultural sectors, similar to others, is daily. To the best of our knowledge, there are no studies on the consequences related to the collaboration of public and private organizations in the co-provision of health and social care services based on different reference sectors. For example, the promotion of art therapy in museum is extensively studied (45–47). However, the engaged public and/or private cultural organizations are considered exclusively as hosts of art therapy programs and are not integrated in the decision-making. This research has the purpose to investigate the outputs of the collaboration between public and private organizations of different sectors in the joint implementation of art therapy programs in terms of economic sustainability and the quality improvement. The object of the study is the Alzheimer Café in Prato, where the provision of art therapy programs is the co-responsibility of a network composed by public and private organizations of the health and social care and cultural sectors.

## 2 Materials and methods

The methodology of the case study was considered the most suitable for pursuing the goal of this research (48, 49). The case study is represented by Alzheimer Café in Prato (Italy). This experience is relevant because here art therapy is a means to create a wider collaboration between partners of different legal nature (public and private) and sectors (health, social and cultural) in a joint decision-making. The case analysis was conducted in three phases:

- *Within case analysis.* Data from each organization involved in the selected case study were acquired through prior documentary analysis of primary and secondary sources. It offered an overview on their governance model and management (50).
- *Data acquisition.* The operative information about the co-provision of art therapy was collected by a focus group including professionals. The research group invited potential participants to join the online focus group via email. The focus group was chosen for the own high sensitivity and specificity for the collection of qualitative data (51, 52). In terms of choosing a focus group with a limited number of people, it was concluded that six members can represent the organizational dimension of their organizations. Each organization has a plurality of activities executed in the field of action of art therapy (involvement of more than 800 old people, who are hosted in nursing homes, participate in day care centres, or receive some domiciliary services assisted by the two health and social care consortia or the thousands of visitors of the three involved museums). The focus group was conducted online to limit as much logistical and temporal issues as possible to the participants. Before the start of the online meeting, each participant was required to sign a privacy policy document. The gathered research group collected responses (M.G. - focus group’s master and N.P. - observer). The focus group was audio-recorded with the respondents’ consensus and then transcribed. Before the analysis of the collected data, the focus group’s transcription was sent to all participants for the validation of the rightness of the contents. None of the participants suggested any correction or change.
- *Data analysis.* Coding method was used for analyzing qualitative data of focus group and synthetizing them (53–55). Four reference categories were applied as coding categories according to the main variables that influence the co-provision of art therapy into collaboration: (I) professionals; (II) experience; (III) intersectoral public-private collaboration; and (IV) future challenges (Table 1).

**Table 1:** Focus group questions.

I. Professionals	II. Experience	III. Intersectoral public-private collaboration	IV. Future challenges
a. Presentation of participants’ professional profile, current role, work experience, and work context b. Have you had specific training about art therapy or have you trained yourself on-the-job?	c. What activity do you carry out together? d. With how many people have you carried out art therapy activities over time? e. How would you plan and apply an art therapy program for the elderly, especially with Alzheimer’s disease, and for their caregivers?	f. How much is the synergy between public and private organizations important? g. What are the advantages associated with your private nature? What are the issues? h. What are the opportunities of working in the health and social sectors where the presence of private organizations is becoming much more prevalent than that of public organizations? What are the threats? i. On the other hand, what are the benefits and difficulties of being public?	j. What are your prospects for growth in the field of art therapy, guaranteeing the sustainability of the service?

Focus group’s members were selected for their professional experience and their then-current role in each organization employed in the considered case study and not as a patient or a member of a sample. They subscribed the privacy policy document to consent the management of their personal data in compliance with the European (Regulation (EU) 2016/679, Regulation (EU) No. 536/2014) and national regulation (Italian Law 2019/2017). The request for the approval of research by the ethics committee or the institutional board was not required because of the absence of sensitive health data related to medical treatment and of any research involving human participants (56, 57).

## 3 Results

### 3.1 Case study

The object of the case study was the Alzheimer Café in Prato (Italy). In 1997, the Alzheimer Café were ideated by psychogeriatrician Bere Miein in Leiden (Netherlands) for people in the first stages of the Alzheimer’s disease. The Alzheimer Café was conceptualized as an informal, deinstitutionalized space. At this Café, people with Alzheimer’s disease meet people with a same or similar condition, finding mutual support in their struggle against the illness and loneliness (58). Greater knowledge of their own condition reduces anxiety, stress, and a sense of shame (59). In recent years, Alzheimer Café have spread all over the world due to the effectiveness of this method. In Alzheimer Café, recreational activities are carried out for stimulating memory, supporting psycho-physical wellbeing, and socialisation. These activities have always included non-pharmacological approaches that have often taken the form of arts-based programs (60).

**Figure 1:**
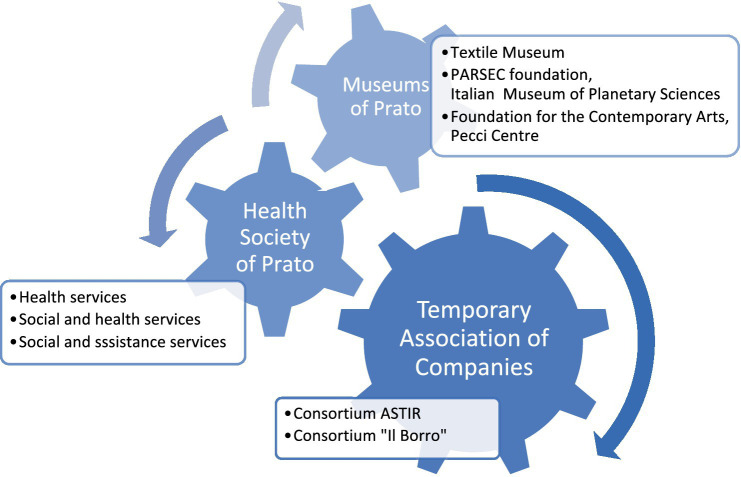
Intersectoral public-private collaboration deputed to art therapy into the Alzheimer Café in Prato.

In 2019, the Alzheimer Café was designed, implemented, and managed by a temporary association of enterprises in Prato. The temporary association of enterprises has been composed by two health and social care consortia (Astir consortium and Il Borro consortium) in collaboration with the Health Society of Prato.^1^ The Alzheimer Café was financed by the Health Society of Prato with €21,000.00 and resources were received from the Tuscany Region for the provision of health and social care services. The Health Society of Prato also made available a location for the provision of the Alzheimer Café. The temporary association of enterprises in collaboration with the three museums of Prato (the Textile Museum,^2^ the PARSEC Foundation, Museum of Planetary Sciences^3^ and Foundation for Contemporary Arts Luigi Pecci Centre^4^) had deputed to co-provide an art therapy program at the Alzheimer Café since 2019 (see Figure 1). Art therapy sustains the integration of health and social responses and taking care of the users, i.e., older people in the first stages of Alzheimer’s disease, by enabling them to be part of the entire society.

At the end of the first year of the art therapy program at the Alzheimer Café, it was decided that the temporary association of enterprises in collaboration with the three involved museums would continue this art therapy program owing to the excellent results achieved, despite the absence of regional funding. This program was directly financed by the abovementioned organizations with their resources from 2020 to 2022.

In 2023, the Health Society of Prato had received a new regional funding to finance a laboratory (€120,000.00), which permits the activation of Alzheimer Café in Prato for the following 2 years (January 2023–December 2024), the provision of the Café at one more location for a better coverage of the territory (in the North and in the South of Prato), and the increase of weekly accesses (from 3 to 5) to the same number of users due to a much more conspicuous nature of the resources.

### 3.2 Focus group data

#### 3.2.1 Professionals

Participants to focus group were three operators of the Alzheimer Café (D.S., coordinator; F.V., professional educator; and S.M., health and social worker) and three educators of the museums (I.I., art historian, coordinator of the educational department of the Foundation for Contemporary Arts Luigi Pecci Centre; S.C., astrophysicist, responsible of the educative and informative activities of the PARSEC Foundation and Museum of Planetary Sciences; F.S., art historian, responsible of the educative service of the Textile Museum of Prato).

The health and social care professionals have not received any specific training on art therapy. They have acquired their competencies on this subject directly on the job or by borrowing skills obtained from other courses (for example, a course on communication with persons with dementia or a course for professional geriatric educators). On the other hand, museum educators received a specific training on art therapy and on the implementation of related programs. Since 2017, indeed, the Tuscany Region has promoted a training course free of charge for museum educators as part of the regional project “Tuscan System for Alzheimer Museums”^5^, which is dedicated toward the acquisition of specific skills for the reception, integration, and inclusion of people with Alzheimer’s disease in museum contexts by museum educators.

#### 3.2.2 Experience

From 2019 to date, the Alzheimer Café in Prato followed up 26 older people with Alzheimer’s disease, divided them into two groups for meeting three times a week. Albeit with a slight decrease in the number of participants (between five and eight in each group), Alzheimer Café continued during the COVID-19 pandemic. The face-to-face meetings were replaced with online meetings and telephone meetings, according to the specific communication skills and needs of the attended participants.

The interventions in the art therapy program were not limited to the organization of practical laboratories; however, attention should be paid to ensure that everyone can enjoy art.

The art therapy program in the case study has been composed by the collection of multiple interventions (for example, the adoption of multisensorial explorative paths for the fruition of multiple kinds of art), each one tailored on every user’s needs (i.e., those who are visually impaired, those who are deaf, those with reduced mobility, those of different ages, or those from different social backgrounds). In this way, the enjoyment of art has been offered to everybody by the breaking down of all possible barriers that could limit art fruition.

#### 3.2.3 Intersectoral public-private collaboration

The added value of Alzheimer Café in Prato is due to the co-design and co-provision of the art therapy program by the intersectoral public-private collaboration.

The collaboration began with the co-financing of an art therapy program for guaranteeing the economic sustainability of the service. The integration of different reference networks has offered major possibilities of fundraising (other public announcements, different type of resources, both services and goods, distinctive professional profiles, etc.), enforcing more and more the economic sustainability of the art therapy service.

Over time, the collaboration has evolved in the sharing of not only economic resources but also ideas, time, spaces, professionals, and reference networks, thereby strengthening the mutual knowledge. It has favoured the co-design and co-responsibility for the provision of art therapy in Alzheimer Café. It improved the service quality in terms of achieved health outcomes. These results were certificated by the psychologist who recognized and reported the effectiveness of the personalized pathway into art therapy program (reduction of anxiety, greater willingness to socialize, active participation of the proposed activity, effective management of symptoms such as psychosis, wandering, restlessness, etc.) and by users’ satisfaction (persistence of the desire to participate and high scores on the satisfaction questionnaire filled both by users and caregivers at home). Positive results were achieved owing to the optimisation of the delivery process of service: no turnover of personnel; progressive extension of the offer; widening of the available places; adoption of always more comfortable and safe locations for the everyday conduction of the Café; and involvement of qualified collaborators.

Learning constantly from past experiences through the application of rigorous evaluation and control mechanisms was possible with the help of performance-oriented strategies and the progressive adaptation of the route to the emerging needs. This approach is peculiar of the private sector and seldom detectable in the public sector. In the intersectoral public-private collaboration, these strategies were borrowed and applied in public organizations (61). At the same time, the public-private collaboration enforced the leader position of the private sector in the competitive market. It was demonstrated by the objective and verified increase of the attributed scores during the selection of other public and private calls for proposals.

Nevertheless, the public sector continued to impose on all network the respect of stringent bureaucratic and only formal constraints, placing limits and clauses even in the face of years of excellent results. In this way, the public sector is still anchored to its old role of a mere funder and controller.

#### 3.2.4 Future challenges

In the future, the intersectoral public-private collaboration should has as its main purpose the development of a specific art therapy program that will take care of caregivers also. Caregivers often feel resigned to no longer living their full life due to being completely immersed in the assistance of a person with Alzheimer’s disease. Caregiver stress can lead to caregivers putting themselves at risk of getting sick. Instead, they should preserve their identity and their physical and mental wellbeing perhaps by attending to an art therapy program.

**Figure 2:**
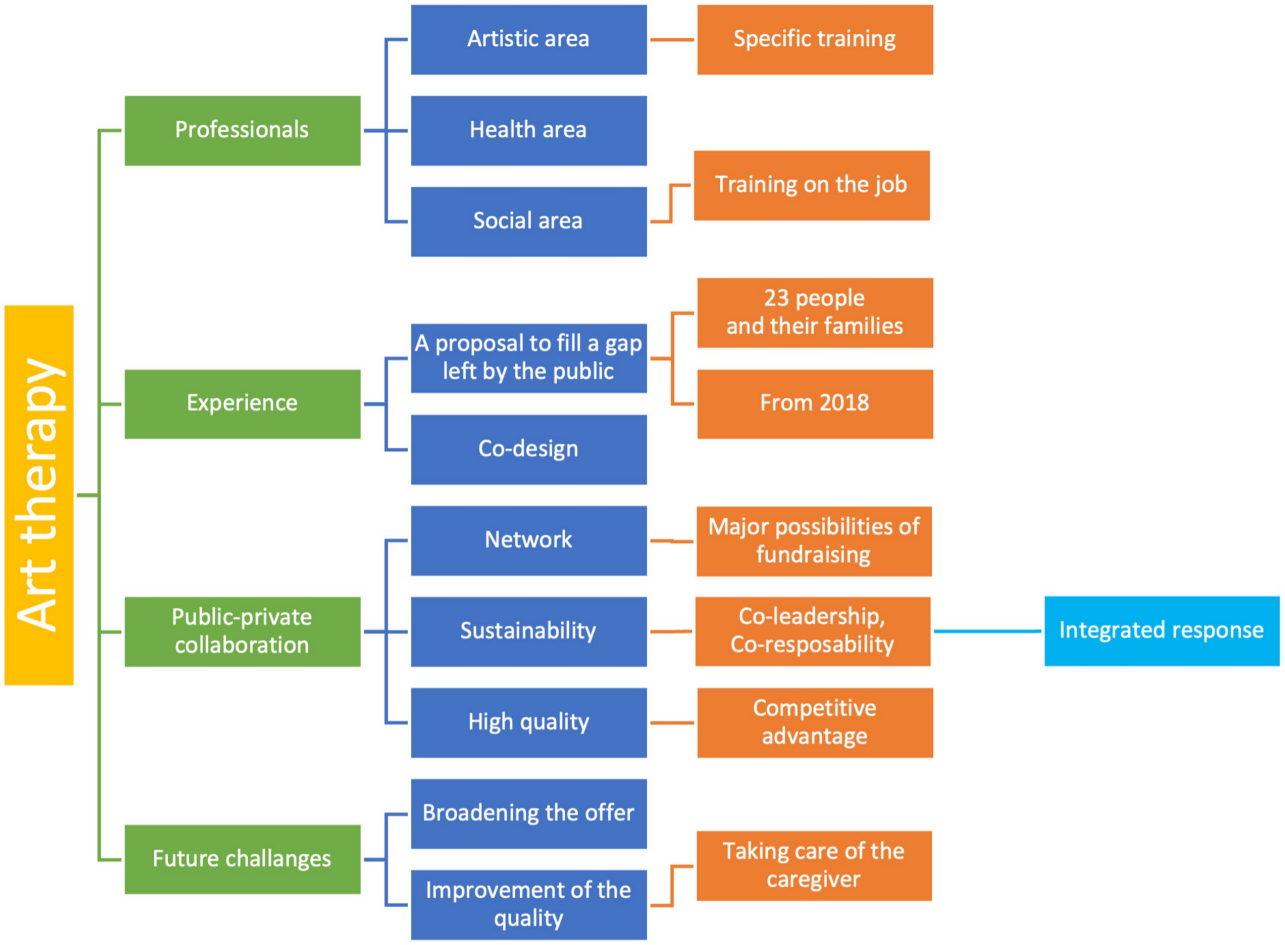
Coding three of focus group’s information.

The intersectoral public-private collaboration should include all public institutions, no profit organizations, and families for the purpose of engaging all stakeholders in order to efficiently execute this program (Figure 2).

## 4 Discussion

Although the first art therapy courses could be dated back to the early 1970s (1, 5), these courses are, at present, still exclusive for psychologists and psychoanalysts or are organized separately for each profile involved in art therapy. The absence of integrated professional training on that topic, especially for health and social care professionals and museum educators, severely limited the matching of supply and demand. The joint training of these two categories probably will resolve the current issues and catalyze the implementation of the art therapy program. In the case study, the intersectoral public-private collaboration was proposed as a concrete solution in order to provide over time an art therapy program that satisfies the emerging needs of this specific target in terms of memory stimulation (19, 20), psycho-physical wellbeing (58, 59), and socialization (22, 23). The implementation of this organizational model was the starting point for the passage from a give-and-take relationship among the public and private organizations involved in the provision of art therapy to the process of co-design, co-provision, and co-responsibility in the results achieved by means of art therapy (41–44).

The added value of the intersectoral public-private collaboration was the mutual support among public and private, health and social care, and cultural organizations. The mutual support was a solution to the resource shortage and the inadequacies in order to keep up with the complex requirements settled on health and social care services provision, i.e., quality improvement, affordability, economic sustainability, budgeting or introduction of evaluation, and control mechanisms (62, 63). The intersectoral public-private collaboration as organizational model can be reproposed in each context where a plurality of subjects, public and private, based on different reference sectors, whether structured or not, want, in general, to collect their resources to invest them in the joint taking care of a frail target.

Putting together their professionals, well-equipped locations, resources, and ideas, each member of the intersectoral public-private collaboration is relying on the resources of the other partner as, otherwise, only their resources would not be enough (64).

In this way, the case study was unknowingly experimenting the implementation of the personal health budget, having already created the needed organizational and managerial substrates for its application (65). The personal health budget is an accounting tool that consents the anticipated determination of the quantity, the type, and the quality of economic, professional, and structural resources that public institutions, no profit organizations (both public and private), and community and personal network should be made available for the joint supply of health and social care pathways for a person with health and/or social complex needs (66, 67).

This tool was created to finance the health and social care pathways of persons with disabilities according to their needs (68, 69). In 2022, in Italy, where the object of the case study is located, the Ministerial Decree No. 77/2022 (70) identified this tool for the economic coverage of all social and health care pathways of frail persons in accordance with their risk level. Despite the strong political mandate and endorsement for the adoption of personal health budget, its use is still contained. An issue for the wider adoption of personal health budget is the absence of an organizational model that creates pre-conditions for the integration of resources. The intersectoral public-private collaboration can, indeed, support this process by proposing first an organizational model that allow for and favor the integration of resources and, as said, something more.

The organizations in the public-private collaboration are applying for having in mid-long future a new role in the community as promoters of pathways for the humanisation of care by means of art (14) and for the population healthy ageing (26, 71, 72). They are working for the promotion of wellbeing and wellness in the society (12, 73). According to these future objectives, the commitment for the next short future of the intersectoral public-private collaboration, such as the case study, is taking care of caregivers also. It must be considered a preventive intervention against the possible onset both of physical and mental diseases. The reduction of the stress and anxiety levels in these persons fully dedicated to the assistance of patients can limit the probabilities of future illness (74–76).

## 5 Conclusions

The intersectoral public-private collaboration for the co-design of health and social care services, especially non-pharmacological approaches such as art therapy, is an effective organizational model. In this context, the integrated use of the different economic, organizational, professional, and structural resources made available by each organization improved safety, quality, and economic sustainability. The achievement of these goals was possible because public and private organizations based on different reference sectors operated all together as a single entity, sustaining co-design, co-funding, and co-provision of inclusive services.

The implementation of a personal health budget is proposed as an operative tool to start and consolidate the integration both of ideas, projects and, only at the end, resources, having an approach of co-leadership and co-responsibility.

The peer-to-peer intersectoral public-private collaboration for the provision of these kinds of service fully satisfies the needs of social integration, acceptance, and cohesion of frail target into the society, as older patients with Alzheimer’s disease and their caregivers in the selected case study, the Alzheimer Café of Prato.

The intersectoral public-private collaboration can be reproposed in every context, in which the shortage of resources and the insufficient quality levels are not guaranteeing over time the provision of integrated and innovative health and social care services referring to non-pharmacological approaches that are more complex and more expensive.

The limitations of this study are the absence of a comparison with other similar realities for the conduction of a cross-case analysis that could consolidate the generalization of the obtained results. Possible developments of this research could be the development of a new study that compares the organizational model adopted for non-pharmacological approaches’ provision in multiple Alzheimer Cafés as a result to their spreading all over the world. It could be also interesting to consider the influence of various national financing, normative, and managerial field of action in the replication of the proposed intersectoral public-private collaboration.

## Data availability statement

The original contributions presented in the study are included in the article, further inquiries can be directed to the corresponding author.

## Ethics statement

Ethical review and approval was not required in accordance with local and national guidelines. Written informed consent to participate in this study was provided by the participants.

## Author contributions

MG: conceputualization, data curation, writing-original draft preparation, visualization, and project administration. NP: resources and supervision. MG and NP: methodology, formal analysis, investigation, writing-review and editing. All authors contributed to the article and approved the submitted version.

## Footnotes

^1^The Health Society is a public consortium established between the municipalities of the province of Prato and the Local health Authority USL Toscana Centro. It is an organisational model for the government of the health, social, and welfare services of the territory, introduced by Regional Law no. 40/2005 in order to guarantee more efficient and effective services, high quality of services, universality and equal access to services, proximity and participation of the citizens, and the no profit organizations.

^2^Textile Museum of Prato (https://www.museodeltessuto.it/).

^3^Foundation of Comtemporary Arts Pecci Centre (https://centropecci.it/).

^4^PARSEC Foundation and Museum of Planetary Sciences (https://www.fondazioneparsec.it/it/il-museo-di-scienze-planetarie/).

^5^Tuscan System of Museums for Alzheimer (Chi siamo - Musei Toscani per l’Alzheimer:- https://www.museitoscanialzheimer.org/chi-siamo/).
